# Inhibition of Inflammatory Response by Artepillin C in Activated RAW264.7 Macrophages

**DOI:** 10.1155/2013/735176

**Published:** 2013-05-28

**Authors:** Ewelina Szliszka, Anna Mertas, Zenon P. Czuba, Wojciech Król

**Affiliations:** Department of Microbiology and Immunology, Medical University of Silesia in Katowice, Jordana 19, 41 808 Zabrze, Poland

## Abstract

Artepillin C (3,5-diprenyl-4-hydroxycinnamic acid) is the main bioactive component of Brazilian green propolis. The aim of this study was to investigate the anti-inflammatory effect of artepillin C on LPS + IFN-**γ**- or PMA-stimulated RAW264.7 macrophages. The cell viability was evaluated by MTT and LDH assays. The radical scavenging ability was determined using DPPH^•^ and ABTS^•+^. ROS and RNS generation was analyzed by chemiluminescence. NO concentration was detected by the Griess reaction. The release of various cytokines by activated RAW264.7 cells was measured in the culture supernatants using a multiplex bead array system based on xMAP technology. NF-**κ**B activity was confirmed by the ELISA-based TransAM NF-**κ**B kit. At the tested concentrations, the compound did not decrease the cell viability and did not cause the cytotoxicity. Artepillin C exerted strong antioxidant activity, significantly inhibited the production of ROS, RNS, NO, and cytokine IL-1**β**, IL-3, IL-4, IL-5, IL-9, IL-12p40, IL-13, IL-17, TNF-**α**, G-CSF, GM-CSF, MCP-1, MIP-1**α**, MIP-1**β**, RANTES, and KC, and markedly blocked NF-**κ**B expression in stimulated RAW264.7 macrophages. Our findings provide new insights for understanding the mechanism involved in the anti-inflammatory effect of artepillin C and support the application of Brazilian green propolis in complementary and alternative medicine.

## 1. Introduction

**Figure 1:**
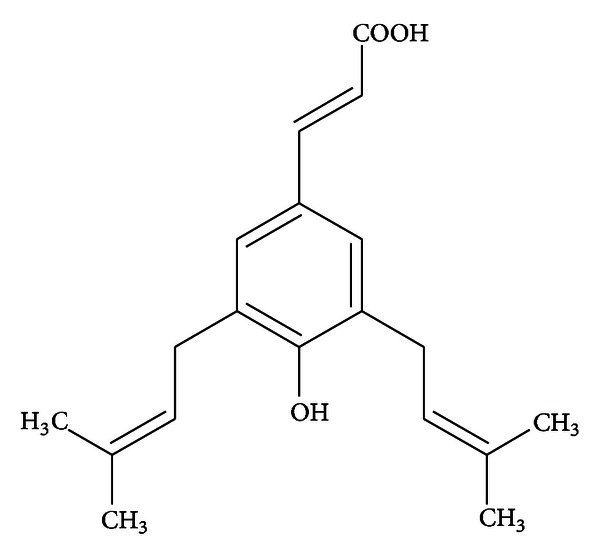
Chemical structure of artepillin C (3,5-diprenyl-4-hydroxycinnamic acid).

Extracts from Brazilian green propolis possess antioxidant, antimicrobial, anti-inflammatory, antiangiogenic, chemopreventive, and anticancer properties [1–7]. The biological and pharmacological activities of propolis are mainly contributed by its phenolic and polyphenolic components [8–11]. Artepillin C (3,5-diprenyl-4-hydroxycinnamic acid) is the major biologically active phenolic ingredient identified in green propolis derived from southeast Brazil [12–14]. Honeybees collect exudates from plant* Baccharis dracunculifolia* in order to produce green propolis, which contains large concentration of this compound [15, 16]. Artepillin C is a simple phenol constructed of single ring with two prenyl groups (Figure 1). Low molecular weight and chemical structure increase its affinity for cell membrane along with incorporation into the cell and block the conversion to an inactive conjugated form during intestinal absorption. These properties suggest high bioavailability and biological activity of artepillin C [17–19].

This study was designed to investigate the anti-inflammatory effect of artepillin C on LPS (lipopolysaccharide) + IFN-*γ* (interferon *γ*) or PMA (phorbol 12-myristate 13-acetate) stimulated RAW264.7 macrophages. Numerous findings confirm that this cinnamic acid derivative, similar to green propolis, exhibits strong antioxidant, immunomodulatory, and anticancer properties [2, 4, 6, 9, 12, 14]. Propolis affects the nonspecific immunity *via* modulation of macrophages activity. During inflammation, macrophages upon interaction with microorganisms produce excessive amounts of mediators such as reactive oxygen (ROS) and nitrogen species (RNS), nitric oxide (NO), and various cytokines. Oxidants and nitrogens generated by macrophages to destroy phagocytized pathogens are also involved in the tissues injury associated with inflammatory process [20–22]. Cytokines secreted by activated macrophages play a significant role in induction and regulation of cellular interactions, but their overexpression causes pathological, acute, or chronic inflammatory responses [20]. Nuclear factor *κ*B (NF-*κ*B) is a critical transcription factor that expresses the genes engaged in inflammation. The main inducible form of NF-*κ*B is a heterodimeric complex consisting of the p50/p65 (protein 50/protein 65) subunits. After activation the NF-*κ*B heterodimer p50/p65 is translocated rapidly from cytoplasm to the nucleus, where it binds to specific DNA motif and modulates the transcription of target genes including proinflammatory cytokines, chemokines, adhesion molecules, and enzymes [23–25].

This is the first comprehensive report explaining anti-inflammatory activity of artepillin C on macrophage *in vitro* model. We determined the effect of artepillin C on production of NO, ROS, RNS, and cytokines: interleukin (IL): IL-1*α*, IL-1*β*, IL-3, IL-4, IL-5, IL-6, IL-9, IL-10, IL-12p40, IL-13, IL-17, TNF-*α* (tumor necrosis factor *α*), IFN-*γ* (interferon *γ*), G-CSF (granulocyte colony-stimulating factor), GM-CSF (granulocyte-macrophage colony-stimulating factor), MCP-1 (monocyte chemotactic protein 1), MIP-1*α* (macrophage inflammatory protein 1*α*), MIP-1*β* (macrophage inflammatory protein 1*β*), RANTES (regulated upon activation of normal T cell expressed and secreted), and KC (keratinocyte-derived chemokine, keratinocyte chemoattractant), inhibition of NF-*κ*B (p65) in stimulated RAW264.7 macrophages. The present study demonstrated the potential of artepillin C in suppressing chronic inflammation and reducing risk of related human health problems. Our findings provide new insights for understanding the mechanism involved in the anti-inflammatory properties of artepillin C and support the application of Brazilian green propolis in complementary and alternative therapies.

## 2. Materials and Methods

### 2.1. General

Artepillin C (3,5-diprenyl-4-hydroxycinnamic acid) was provided by Wako Pure Chemicals (Osaka, Japan) as a natural constituent isolated from Brazilian green propolis. The compound was dissolved in DMSO (dimethyl sulphoxide) to obtain the working concentrations. LPS (LPS *E. coli* O111:B4) was purchased from Fluka Chemie GmbH (Buchs, Switzerland), recombinant mouse IFN-*γ* was purchased from R&D Systems (Minneapolis, MN, USA), DMSO and PMA were purchased from Sigma Chemical Company (St. Louis, MO, USA). 

### 2.2. Cell Culture

Murine peritoneal macrophage cell line RAW264.7 was obtained from ATCC (American Type Culture Collection, Manassas, VA, USA). Cells were cultured in Dulbecco's modified Eagle's medium supplemented with 10% heat-inactivated fetal bovine serum, 100 U/mL penicillin, and 100 *μ*g/mL streptomycin at 37°C and 10% CO_2_ in a humidified incubator [26, 27]. Reagents for cell culture were purchased from ATCC. RAW264.7 cells were seeded at a density of 1 × 10^6^/mL cells (2 × 10^5^/well) in 96-well plates at the presence of LPS (200 ng/mL) and IFN-*γ* (25 U/mL) with or without artepillin C for 24 h.

### 2.3. Cell Viability Assay

The cell viability was determined by the 3-(4,5-dimethyl-2-thiazyl)-2,5-diphenyl-2*H*-tetrazolium bromide (MTT) reduction assay as previously described [28, 29]. This test is based on the cleavage of the tetrazolium salt MTT to a blue formazan dye by viable cells. The RAW264.7 cells (1 × 10^6^/mL) were seeded 4 h before the experiments in a 96-well plate. Artepillin C at the concentrations of 25–100 *μ*M with or without LPS + IFN-*γ* was added to the cells. The final volume was 200 *μ*L. After 24 h the medium was removed, and 20 *μ*L MTT solutions (5 mg/mL) (Sigma Chemical Company, St. Louis, MO, USA) was added to each well for 4 h. The resulting formazan crystals were dissolved in DMSO. The controls included native cells and medium alone. The spectrophotometric absorbance was measured at 550 nm wavelength using a microplate reader (ELx 800, Bio-Tek Instruments Inc., Winooski, VT, USA). The cytotoxicity as percentage of cell death was calculated by the formula (1 − [absorbance of experimental wells/absorbance of control wells]) × 100%.

### 2.4. Cytotoxicity Assay

The cytotoxicity of artepillin C was determined by using LDH activity assay kit (Roche Diagnostics GmbH, Mannheim, Germany) [29, 30]. Lactate dehydrogenase (LDH) is a stable cytosolic enzyme released upon membrane damage in necrotic cells. The RAW264.7 (1 × 10^6^/mL) cells were treated with 25–100 *μ*M artepillin C with or without LPS + IFN-*γ* for the indicated period of time. LDH released in culture supernatants is detected with coupled enzymatic assay, resulting in the conversion of a tetrazolium salt into a red formazan product. The maximal release of LDH was obtained after treating control cells with 1% Triton X-100 (Sigma Chemical Company, St. Louis, MO) for 10 min at room temperature. The spectrophotometric absorbance was measured at 490 nm wavelength using a microplate reader (ELx 800, Bio-Tek Instruments Inc., Winooski, VT, USA). The percentage of necrotic cells was expressed using the following formula: (sample value/maximal release) × 100%.

### 2.5. DPPH Radical Scavenging Activity

Hydrogen-donating activity was measured using 1,1-diphenyl-2-picrylhydrazyl radical (DPPH) (Sigma Chemical Company, St. Louis, MO) following a previously reported protocol [31]. Artepillin C (0.1 mL) was mixed with 0.9 mL of 0.041 mM DPPH^•^ in ethanol and stored at room temperature in the dark for 30 min. The absorbance of the resulting solutions was measured at 517 nm wavelength using V-630 Spectrophotometer (Jasko International Co., Tokyo, Japan). The percentage of scavenging activity was calculated by the formula: DPPH^•^ scavenging activity = 1 − (absorbance of experimental wells/absorbance of control wells) × 100%. The scavenging activity of the sample was expressed as the ED_50_ value, the concentration required to scavenge 50% of DPPH^•^. Ascorbic acid was used as a standard.

### 2.6. ABTS Cation Radical Scavenging Activity

2,2′-Azinobis(3-ethylbenzothiazoline-6-sulfonic acid) radical cation (ABTS^•+^) (Sigma Chemical Company, St. Louis, MO) scavenging activity was determined according to the previously described procedure [31]. Artepillin C (0.1 mL) was mixed with potassium phosphate buffer (0.1 mL of 0.1 M) and hydrogen peroxide (10 *μ*L of 10 mM) and preincubated at 37°C in the dark for 5 min. Next, ABTS (30 *μ*L of 1.25 mM in 0.05 M phosphate-citrate buffer) and peroxidase (30 *μ*L of 1 unit/mL) were added to the mixture and then incubated at 37°C in the dark for 10 min. The absorbance of the resulting solutions was measured at 417 nm wavelength using V-630 Spectrophotometer (Jasko International Co., Tokyo, Japan). The percentage of scavenging activity was calculated by the formula: ABTS^•+^ scavenging activity = 1 − (absorbance of experimental well/absorbance of control wells) × 100%. The scavenging activity of the sample was expressed as the ED_50_ value, the concentration required to scavenge 50% of ABTS^+^. Ascorbic acid was used as a standard.

### 2.7. Detection of ROS and RNS Production by Chemiluminescence

The chemiluminescence of RAW264.7 macrophages was evaluated by microplate method in Hank's balanced salt solution, pH 7.4, at room temperature. The cells were incubated with 0.5–50 *μ*M artepillin C for 30 min. Next, luminol (Sigma Chemical Company, St. Louis, MO, USA) solution was added to wells containing 2 × 10^5^ cells, giving a final concentration of 110 *μ*M. After 5 min, for macrophages stimulation, PMA solution was injected to obtain the concentration of 0.8 *μ*M. The final volume of each sample was 200 *μ*L. The chemiluminescence was determined for 5 min with luminol alone and after stimulation with PMA for 30 min. The measuring system was equipped with LB 960 CentroXS^3^ microplate luminometer (Berthold Technologies GmbH, Wildbad, Germany) [27, 32].

### 2.8. Quantification of NO Production

RAW264.7 macrophages (1 × 10^6^/mL) stimulated with LPS + IFN-*γ* were incubated with 25–100 *μ*M artepillin C for 24 h. After this time, NO production was determined by measuring the accumulation of nitrite, a stable end product, in the culture supernatant according to the Griess reaction [21, 27]. Equal volumes of culture supernatant from each well or medium (100 *μ*L) were mixed with 100 *μ*L of Griess reagent in a 96-well plate and incubated for 15 min at room temperature. The spectrophotometric absorbance was read at 550 nm wavelength in Eon Microplate Spectrophotometer (BioTek, Winooski, VT, USA), and the nitrite concentration in the medium was calculated using sodium nitrite as a standard. Nitrite was not detectable in cell-free medium.

### 2.9. Multiplex Bead-Based Cytokine Assay

Cytokines released from RAW264.7 macrophages treated with artepillin C were determined in the cell culture supernatants with a Pro Mouse Cytokines 20-plex assay kit for IL-1*α*, IL-1*β*, IL-3, IL-4, IL-5, IL-6, IL-9, IL-10, IL-12p40, IL-13, IL-17, TNF-*α*, IFN-*γ*, G-CSF, GM-CSF, MCP-1, MIP-1*α*, MIP-1*β*, RANTES, and KC (Bio-Rad Laboratories Inc, Hercules, CA, USA). This assay was performed using Bio-Plex 200 System based on xMAP suspension array technology (Bio-Rad Laboratories Inc, Hercules, CA, USA). The LPS + IFN-*γ* stimulated and native (control) RAW264.7 cells (1 × 10^6^/mL) were incubated with or without 50–100 *μ*M artepillin C for 24 h. Standard curves for each cytokine were generated using kit-supplied reference cytokine sample. The assay is designed for the multiplexed quantitative measurement of multiple cytokines in a single well using 50 *μ*L of sample. Briefly, the following procedure was performed: after pre-wetting the 96-well filter plate with washing buffer, the solution in each well was aspirated using a vacuum manifold. Next, the cell culture supernatants were incubated with antibody-conjugated beads for 30 min. Following the incubational period, detection antibodies and streptavidin-PE were added to each well for 30 min. Then, after washing with buffer to remove the unbound streptavidin-PE, the beads bound to each cytokine were analyzed in the Bio-plex Array Reader (Bio-Plex 200 System). The fluorescence intensity was evaluated using Bio-Plex Manager software (Bio-Rad) [27, 33].

### 2.10. The Activity of NF-*κ*B

NF-*κ*B activity was measured by the ELISA-based TransAM NF-*κ*B kit (Active Motif Europe, Rixensart, Belgium) in nuclear extract. RAW264.7 (1 × 10^6^/mL) cells were seeded in Petri dishes 4 h before the experiments, and then incubated with 50–100 *μ*M artepillin C with or without LPS + IFN-*γ* for 4 h. The nuclear extracts were prepared using Nuclear Extract kit obtained from Active Motif Europe (Rixensart, Belgium). The TransAM NF-assay for NF-*κ*B (p65) activity was performed according to the vendor's protocol [24, 25]. NF-*κ*B DNA-binding activity was assessed using the ELISA kit for the transcription factor p65. Oligonucleotides containing the NF-*κ*B consensus binding site (5′-GGGACTTCC-3′) were immobilized on a 96-well plate. The active forms of NF-*κ*B in the nuclear extracts were bound to the oligonucleotides on the plate and detected colorimetrically. The samples were read at an absorbance of 450 nm on a V-630 Spectrophotometer (Jasko International Co., Tokyo, Japan) with a reference wavelength of 650 nm.

### 2.11. The Statistical Analysis

The values represent mean ± SD of two, three, or four independent experiments performed in duplicate or quadruplicate. Significant differences were analyzed using Student's *t*-test, and *P* values < 0.05 were considered significant. The concentration-response curves were analyzed using Pharma/PCS version 4 (Pharmacological Calculations System) software.

## 3. Results

### 3.1. Effect of Artepillin C on Viability of RAW264.7 Macrophages

**Figure 2:**
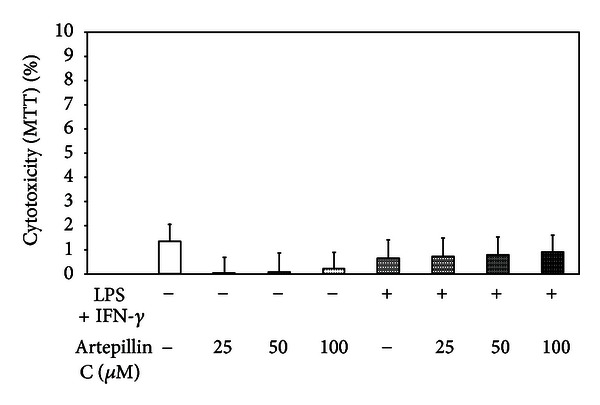
Effect of artepillin C on viability of RAW264.7 macrophages. The cytotoxicity was evaluated by MTT assay after 24 h incubation of RAW264.7 cells with 25–50 *μ*M artepillin C and/or LPS + IFN-*γ*. The values represent mean ± SD of three independent experiments (*n* = 12).

The cell viability in the presence of 25–100 *μ*M artepillin C and/or LPS + IFN-*γ* for 24 h was measured by MTT test (Figure 2). The cytotoxicity of the compound at the same concentrations and incubation time was evaluated by LDH assay. Artepillin C at the concentrations of ≤100 *μ*M did not influence the cell viability and did not exert cytotoxic effect. Therefore, for further studies of anti-inflammatory properties, artepillin C was used at the concentrations of 0.5–100 *μ*M.

### 3.2. Antioxidant Activity of Artepillin C

Antioxidant activity of Artepillin C was investigated by using two different methods for stable DPPH^•^ and ABTS^•+^. The compound exhibited strong scavenging potential against DPPH^•^ (ED_50_ of 24.6 *μ*M) and ABTS^•+^ (ED_50_ of 19.5 *μ*M) compared with ascorbic acid (ED_50_ of 89.8 *μ*M and 57.5 *μ*M, resp.).

### 3.3. Effect of Artepillin C on ROS and RNS Production in PMA-Stimulated RAW264.7 Cells

**Figure 3:**
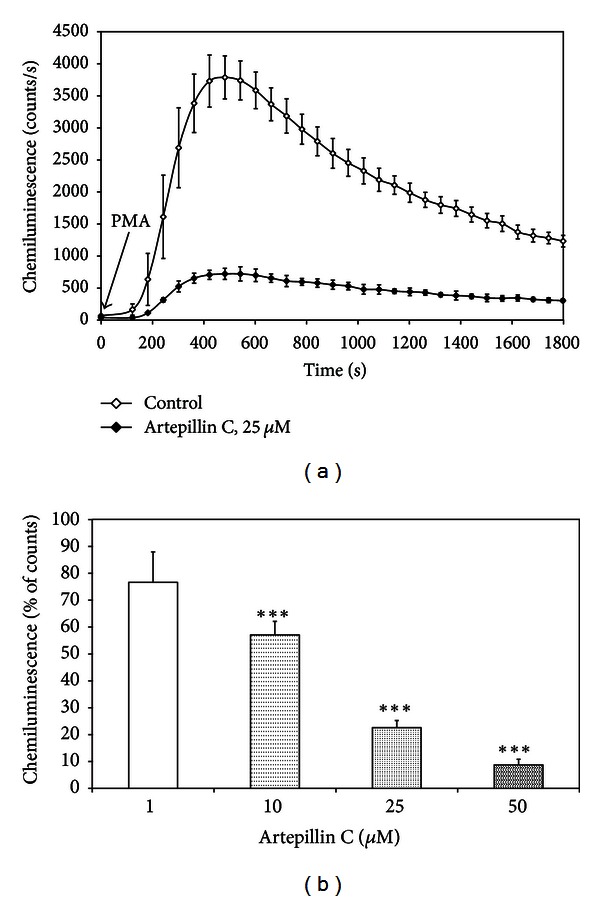
Effect of artepillin C on chemiluminescence of PMA activated RAW264.7 macrophages: (a) time course of chemiluminescence, (b) chemiluminescence of PMA activated RAW264.7 macrophages treated with 1–50 *μ*M artepillin C for 30 min. Chemiluminescence was determined using microplate luminometer and expressed as a percentage of PMA-stimulated cells. The values represent mean ± SD of four independent experiments (*n* = 8) ***P < 0.001 compared to PMA-stimulated cells.

Chemiluminescence was used as an indicator for the production of ROS and RNS in macrophages activated by PMA as a stimulant of protein kinase C (PKC). Artepillin C at the concentrations of 1–50 *μ*M suppressed the chemiluminescence in PMA-treated RAW264.7 cells in dose-dependent manner with an ED_50_ of 7.32 *μ*M (Figure 3).

### 3.4. Effect of Artepillin C on NO Production in LPS + IFN-*γ*-Stimulated RAW264.7 Cells

**Figure 4:**
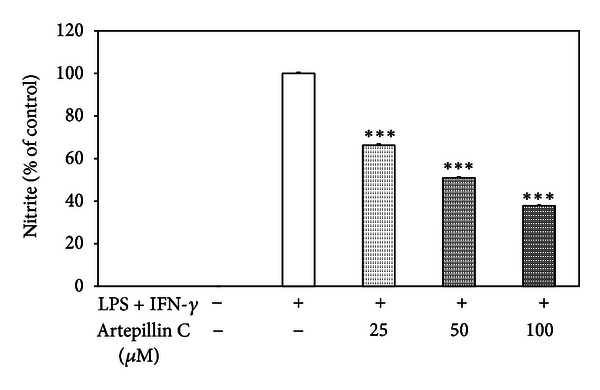
Effect of artepillin C on nitrite (NO) production in LPS + IFN-*γ* stimulated RAW264.7 macrophages. RAW264.7 cells were incubated with of 25–100 *μ*M artepillin C and/or LPS + IFN-*γ* for 24 h. NO production was measured by the Griess reaction assay and expressed as a percentage of LPS + IFN-*γ*-stimulated cells. The values represent mean ± SD of three independent experiments (*n* = 12) ***P < 0.001 compared LPS + IFN-*γ*-stimulated cells.

NO production was determined by measuring the accumulation of nitrite in the culture supernatants using Griess reagent. Macrophages were treated with 25–100 *μ*M artepillin C and/or LPS + IFN-*γ* for 24 h. After LPS + IFN-*γ* stimulation nitrite, concentration markedly increased, but LPS + IFN-*γ*-induced NO synthesis by RAW264.7 cells was significantly decreased by artepillin C in dose-dependent manner (ED_50_ = 53.6 *μ*M). The inhibitory effect of artepillin C on NO production in LPS + IFN-*γ*-stimulated macrophages is presented in Figure 4. The compound did not interfere with the viability of RAW264.7 cells, as shown in MTT and LDH tests. The ED_50_ value of the artepillin C within the nontoxic concentration range suggests that the inhibition of nitrite accumulation was specific to responses by macrophages (due to inhibitory activity on NO production and not cytotoxic property of artepillin C).

### 3.5. Effect of Artepillin C on Cytokine Production in LPS + IFN-*γ*-Stimulated RAW264.7 Cells

**Figure 5:**
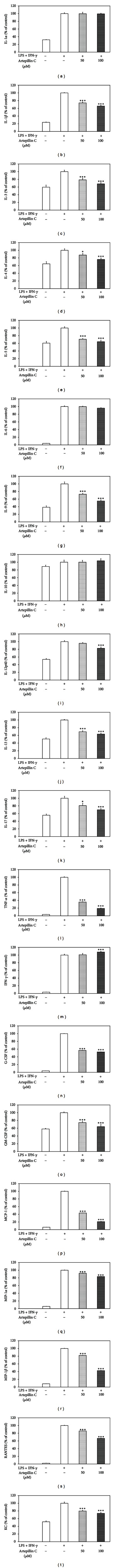
Effect of artepillin C on cytokines production in LPS + IFN-*γ* stimulated RAW264.7 macrophages: (a) IL-1*α*, (b) IL-1*β*, (c) IL-3, (d) IL-4, (e) IL-5, (f) IL-6, (g) IL-9, (h) IL-10, (i) IL-12p40, (j) IL-13, (k) IL-17, (l) TNF-*α*, (m) IFN-*γ*, (n) G-CSF, (o) GM-CSF, (p) MCP-1, (q) MIP-1*α*, (r) MIP-1*β*, (s) RANTES, (t) KC. RAW264.7 cells were incubated with of 50–100 *μ*M artepillin C and/or LPS + IFN-*γ* for 24 h. Cytokine concentrations in the culture medium were determined by Multiplex (20-plex) bead-based cytokine assay. The values represent mean ± SD of two independent experiments (*n* = 8) *P < 0.05, **P < 0.01, ***P < 0.001 compared to LPS + IFN-*γ*-stimulated cells.

The effect of artepillin C on production of cytokine IL-1*α*, IL-1*β*, IL-3, IL-4, IL-5, IL-6, IL-9, IL-10, IL-12p40, IL-13, IL-17, TNF-*α*, IFN-*γ*, G-CSF, GM-CSF, MCP-1, MIP-1*α*, MIP-1*β*, RANTES, and KC in LPS + IFN-*γ*-stimulated RAW264.7 cells is shown in Figure 5. Specifically, native and activated RAW264.7 macrophages were treated with 50–100 *μ*M artepillin C for 24 h. The cytokines released in culture supernatants were analyzed simultaneously by Bio-plex Suspension Array System. This assay is designed for the multiplexed quantitative measurement of multiple cytokines (20-plex) in a single well using 50 *μ*L of sample. Artepillin C significantly decreased IL-1*β*, IL-3, IL-4, IL-5, IL-9, IL-12p40, IL-13, IL-17, TNF-*α*, G-CSF, GM-CSF, KC, MCP-1, MIP-1*α*, MIP-1*β*, and RANTES, but increased IFN-*γ* synthesis in LPS + IFN-*γ*-stimulated RAW264.7 cells in dose-dependent manner. The compound did no influence the concentrations of IL-1*α*, IL-6, and IL-10 in culture supernatants derived from activated macrophages.

### 3.6. Effect of Artepillin C on NF-*κ*B Activity in LPS + IFN-*γ*-Stimulated RAW264.7 Cells

**Figure 6:**
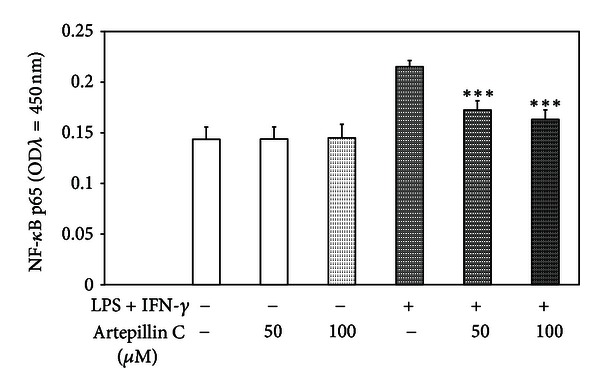
Effects of artepillin C on NF-*κ*B activity in LPS + IFN-*γ* stimulated RAW264.7 macrophages.RAW264.7 cells were incubated with 50–100 *μ*M artepillin C and/or LPS + IFN-*γ* for 4 h. NF-*κ*B (p65) binding activity in nuclear extracts were measured using the ELISA-based TransAM NF-*κ*B assay. The values represent the mean ± SD of three independent experiments (*n* = 6) ***P < 0.001 compared to LPS + IFN-*γ*-stimulated cells.

It has been reported that the transcriptional upregulation of inflammatory mediators is due to NF-*κ*B. The effect of 50–100 *μ*M artepillin C on NF-*κ*B activation in LPS + IFN-*γ*-stimulated RAW264.7 cells was determined by the binding activity of the p65 subunit in nuclear extracts using the ELISA-based TransAM NF-*κ*B test. The incubation of stimulated macrophages with 50–100 *μ*M artepillin C for 4 h markedly blocked the NF-*κ*B activation in dose-dependent manner compared with that of LPS + IFN-*γ*-treated RAW264.7 cells (Figure 6). 

## 4. Discussion

Natural products are promising source for the discovery of new pharmaceuticals. Propolis is a complex mixture of substances collected by honeybees from exudates of various plants. The biologically active molecules in green propolis are flavonoids, phenolic acids, and their esters present in resin [10, 11, 34]. These components act as scavengers of free radicals and inhibitors of nitric oxide and inflammatory cytokines production by neutrophils and/or macrophages [1, 3, 4, 13, 26]. Green propolis from southern region of Brazil is rich in prenylated derivatives of cinnamic acid, such as artepillin C [15, 16]. Several observations suggest the important role of artepillin C in immunoregulation [35, 36].

Brazilian green propolis exerts antioxidant properties by inhibiting chemiluminescence reactions and scavenging ROS. The topical or oral treatment of animals with Brazilian propolis extracts demonstrates potential effect against oxidative stress [37]. Ahn et al. proved that phenolic and polyphenolic compounds contribute to the elevated antioxidant and immunomodulatory activities of propolis [9]. Artepillin C prevents oxidative damage and suppresses lipid peroxidation [38]. DPPH^•^ and ABTS^•+^ tests have been widely used for evaluating antioxidant properties of natural phenolic compounds. Antioxidants intercept the free radical chain oxidation by donating hydrogen from the phenolic hydroxyl groups, thereby forming stable end products, which do not initiate or propagate further oxidation. Artepillin C exerted strong free radical scavenging activity based on reduction of DPPH^•^ and ABTS^•+^. Izuta et al. confirmed the ability of scavenging DPPH^•^ by green propolis and artepillin C, in contrast to its other hydroxycinnamic acid derivatives, drupanin and baccharin, which did not exert similar effect. The findings indicate that 3-prenyl chain of cinnamic acid is important for antioxidant activity of artepillin C [39]. In the present study, we demonstrated potent scavenging capability of artepillin C against DPPH^•^ and ABTS^•+^. Regarding the anti-inflammatory property, the effect of artepillin C in ROS and RNS scavenging was also determined by chemiluminescence assay. We showed for the first time the role of artepillin C in the oxidative metabolism of PMA-stimulated macrophages. The ROS and RNS release by activated RAW264.7 cells was significantly inhibited by artepillin C. Simões et al. and Krol et al. described that extracts of Brazilian green and Polish propolis decrease the chemiluminescence produced by stimulated neutrophils [3, 40, 41]. Cinnamic acids derivatives also enhanced the chemiluminescence in PMA-activated neutrophils [3, 40, 42].

Similar to ROS and RNS, NO generated by inducible nitric oxide synthase (iNOS) acts as reactive molecule in host defense against pathogens. However, excessive release of NO has detrimental effects on many organ systems of the body, leading to tissue damage and even to a fatal development such as septic shock [43]. Therefore, the inhibition of NO accumulation mediated by inflammatory stimuli may be beneficial. Song et al. evaluated that treatment of RAW264.7 cells with extract of Korean propolis markedly blocks NO production, iNOS mRNA, and protein expression induced by LPS + IFN-*γ* [44]. Blonska et al. reported the suppression of NO synthesis and iNOS mRNA expression in LPS-stimulated J774A.1 macrophages by extract of Polish propolis and its phenolic components: chrysin, galangin, kaempferol, and quercetin [45]. Paulino et al. demonstrated that artepillin C decreases NO concentration in RAW264.7 cells incubated with LPS [35]. Similar to previous study, our results confirm the effect of artepillin C on NO generation in RAW264.7 cells. 

Macrophages are a major source of many cytokines involved in immune response, hematopoiesis, inflammation, and other homeostatic processes. Upon stimulation by microorganisms, microbial products (for example LPS), or endogenous factors (including cytokines), macrophages synthetize *de novo* and release a large variety of cytokines: IL-1, IL-3, IL-4, IL-5, IL-6, IL-8, IL-9, IL-10, IL-12, IL-13, IL-17, TNF-*α*, IFN-*α*, IFN-*γ*, TGF-*β*, M-CSF, G-CSF, GM-CSF, MCP-1, MCP-3, MCP-5, MIP-1, MIP-2, RANTES, MIF, and KC. In addition, these cytokines can modulate most of the functions of macrophages, cell surface markers expression, and other cytokines secretion. The cytokine network plays a key role in regulation of macrophages activation [46]. Some cytokines (such as IL-3, IFN-*γ*, and GM-CSF) increase the production of various cytokines by macrophages, while others (IL-10, IL-13, and TGF-*β*) inhibit their expression. Chemoattractant cytokines called chemokines (MCP-1, MCP-3, MIP-1, MIP-2, RANTES, and KC) contribute to the recruitment of circulating monocytes within tissues [46, 47]. In the present study we investigated for the first time the influence of artepillin C on production of cytokine IL-1*α*, IL-1*β*, IL-3, IL-4, IL-5, IL-6, IL-9, IL-10, IL-12p40, IL-13, IL-17, TNF-*α*, IFN-*γ*, G-CSF, GM-CSF, MCP-1, MIP-1*α*, MIP-1*β*, RANTES, and KC in LPS + IFN-*γ*-stimulated macrophages. Inflammatory cytokines are synthetized by innate immune cells during infection. LPS induces very strong inflammation, resulting in generation of IL-1, IL-6, IL-12, and TNF-*α* by the macrophages [47]. The upregulation of proinflammatory cytokines in LPS or LPS + IFN-*γ*-stimulated macrophages was blocked by propolis. We showed that artepillin C significantly downregulated the release of IL-1*β*, IL-12p40, and TNF-*α* by LPS + IFN-*γ*-treated RAW264.7 cells, whereas the synthesis of IL-1*α* and IL-6 was not affected. Bachiega et al. described the increase of IL-1*β* and decrease of IL-6 production by Brazilian green propolis extract and its phenolic acids in peritoneal murine macrophages challenged with LPS [48]. Shi et al. and Wang et al. noticed that extracts from Chinese propolis suppress mRNA and protein expression of IL-1*β* and IL-6 induced by LPS in RAW264.7 cells [49, 50]. Blonska et al. reported the inhibition of IL-1*β* mRNA and protein expression in LPS-stimulated J774A.1 macrophages by extract of Polish propolis and its flavones [45]. The secretion of interleukin IL-3, IL-4, IL-5, IL-9, IL-13, and IL-17 but not IL-10 in LPS + IFN-*γ*-activated RAW264.7 cells was also reduced by artepillin C. The *in vivo* experiment performed by Franchin et al. showed downregulation of IL-1*β* and TNF-*α* production by extract of geopropolis from *Melipona scutellari* [51]. In other *in vivo* test Paulino et al. found out that artepillin C significantly inhibited paw edema in carrageenan-induced model of peritonitis through decrease of the number of neutrophils in the peritoneal cavity [35]. The concentration of IFN-*γ* in stimulated RAW264.7 cells treated simultaneously with artepillin C was slightly increased. The colony-stimulating factors, G-CSF and GM-CSF release in activated RAW264.7 macrophages, were decreased by artepillin C. Chemokines are a family of chemotactic cytokines regulating migration and trafficking of inflammatory cells [46]. Our study demonstrated that artepillin C suppresses the expression of MCP-1, MIP-1*α*, MIP-1*β*, RANTES, and KC in LPS + IFN-*γ*-stimulated RAW264.7 cells. These results suggest a significant contribution of artepillin C in the modulation of chemokine-mediated inflammation.

In LPS-induced models, the production of numerous inflammatory mediators by activated macrophages is regulated by NF-*κ*B transcription factor, and these signaling pathways mediating inflammatory responses have been established in many previous reports [23, 44, 52]. Song et al. showed the decrease of NF-*κ*B binding activity by extract of Korean propolis in RAW264.7 macrophages [44]. Wang et al. found that extract of Chinese propolis inhibits the LPS-induced phosphorylation of I*κ*B*α* involved in NF-*κ*B expression in RAW264.7 cells and reduces the TNF-*α*-stimulated activation of NF-*κ*B in human embryonic kidney HEK293 cells [50]. Paulino et al. for the first time proved that artepillin C blocks NF-*κ*B activation in HEK293 cells [35]. Artepillin C has the ability to enhance the inflammatory response and carcinogenesis by targeting NF-*κ*B signaling pathway. In our recent study artepillin C markedly attenuated LPS + IFN-*γ*-induced activation of NF-*κ*B in RAW264.7 cells. Previously we demonstrated the overcome of resistance to TRAIL-mediated apoptosis in cancer cells by artepillin C and Brazilian green propolis extract through suppression of NF-*κ*B activation [5, 14].

Numerous *in vitro *and *in vivo* studies confirm the fact that absorption and bioavailability of artepillin C are sufficient to produce strong biological effects [17, 18, 35]. The findings suggest potential application of Brazilian green propolis rich in artepillin C as a dietary source of anti-inflammatory nutraceuticals used in functional foods and supplemental products. 

## 5. Conclusion

Propolis became a subject of special interest as a source of valuable phenolic and polyphenolic compounds to develop pharmaceuticals or dietary supplements for the prevention or treatment of inflammatory diseases. Artepillin C as a main ingredient of Brazilian green propolis might represent a new class of bioavailable dietary-derived antioxidant with potent anti-inflammatory activity.
